# Supplementary figures and images for: Sequential Data Assimilation of the Stochastic SEIR Epidemic Model for Regional COVID-19 Dynamics
Source: Bull Math Biol. 2020 Dec 8;83(1):1. doi: 10.1007/s11538-020-00834-8 (PMC7721793; doi:10.1007/s11538-020-00834-8)

## Münster

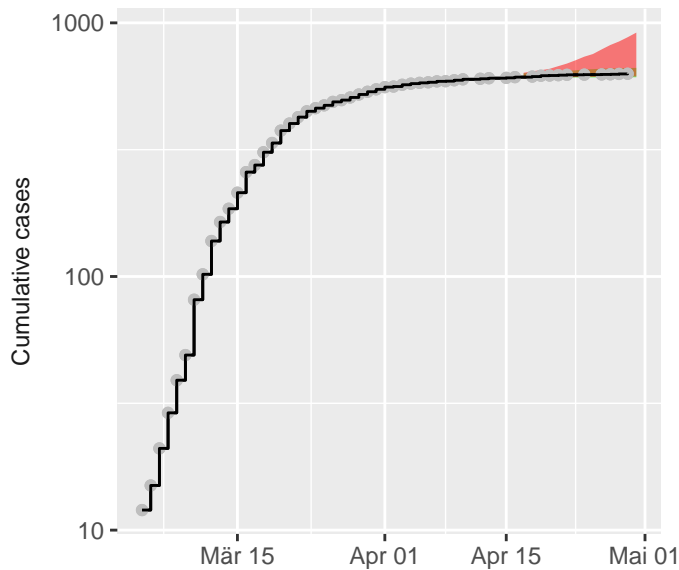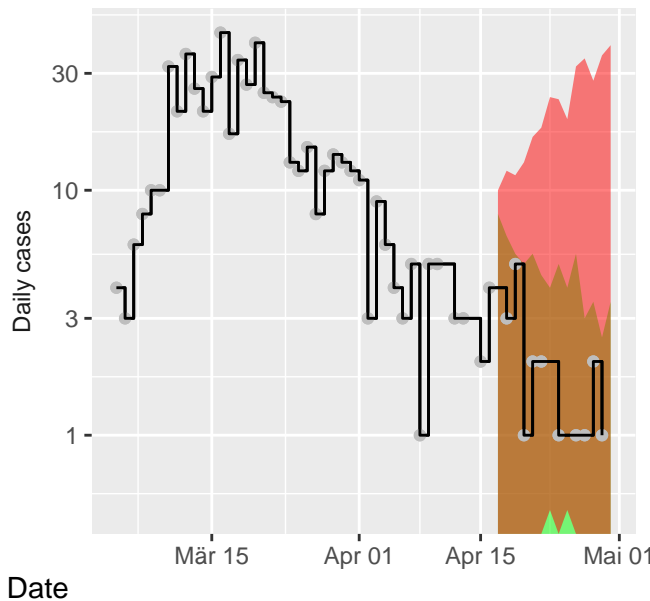

## Göppingen

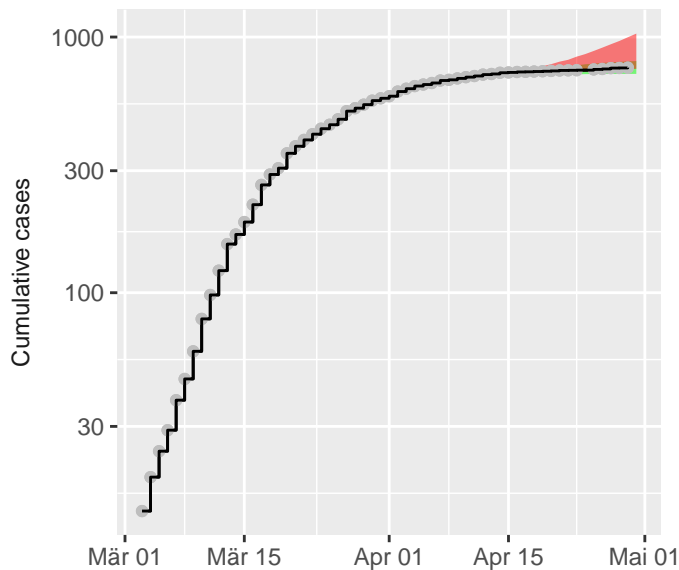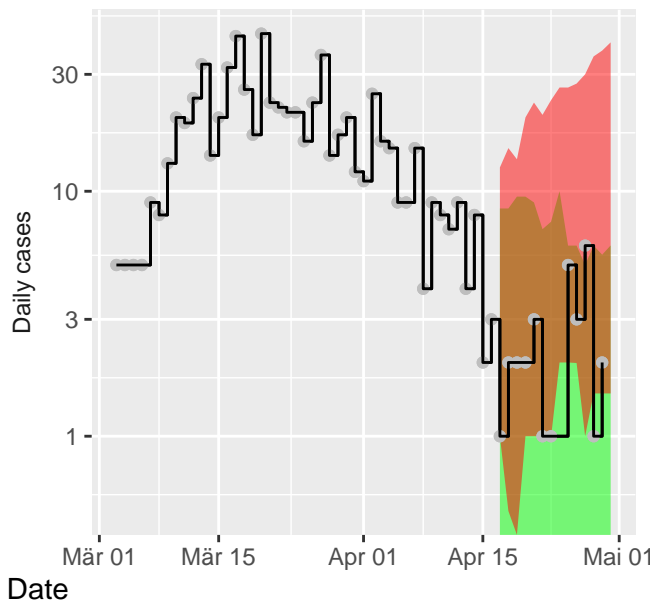

Supplement: Supplementary file 1 — Supplementary material 1 (pdf 10 KB) [file 11538_2020_834_MOESM1_ESM.pdf]

## Städteregion Aachen

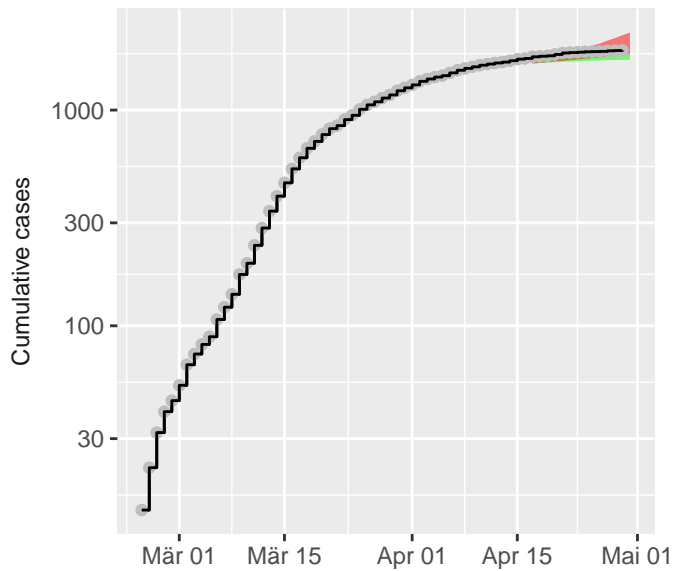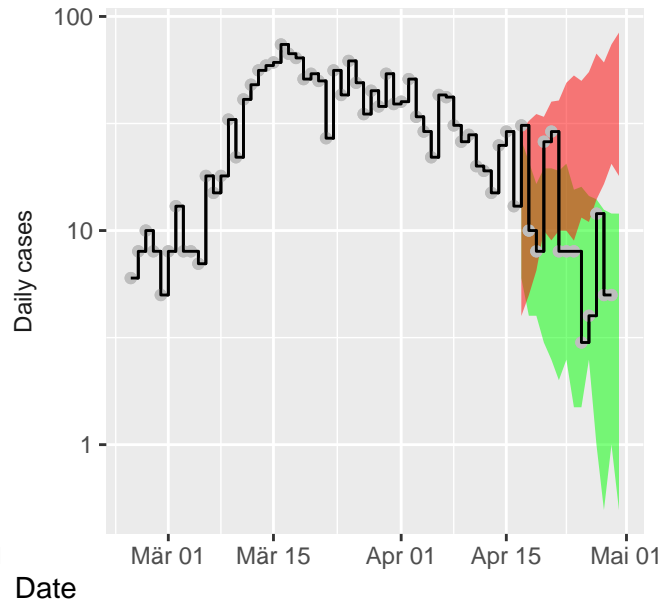

## Wesel

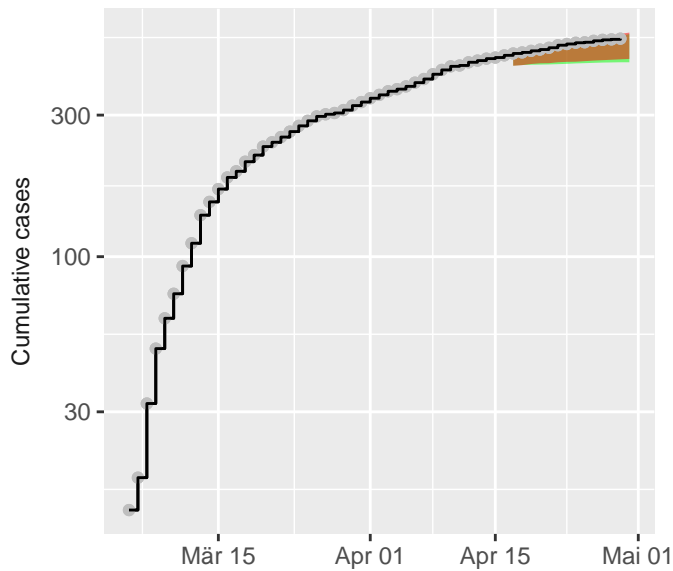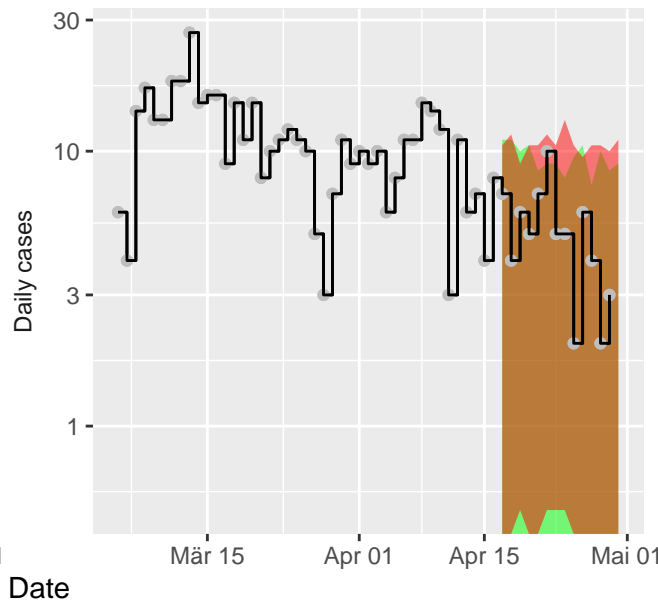

Supplement: Supplementary file 2 — Supplementary material 2 (pdf 11 KB) [file 11538_2020_834_MOESM2_ESM.pdf]

## Schwäbisch Hall

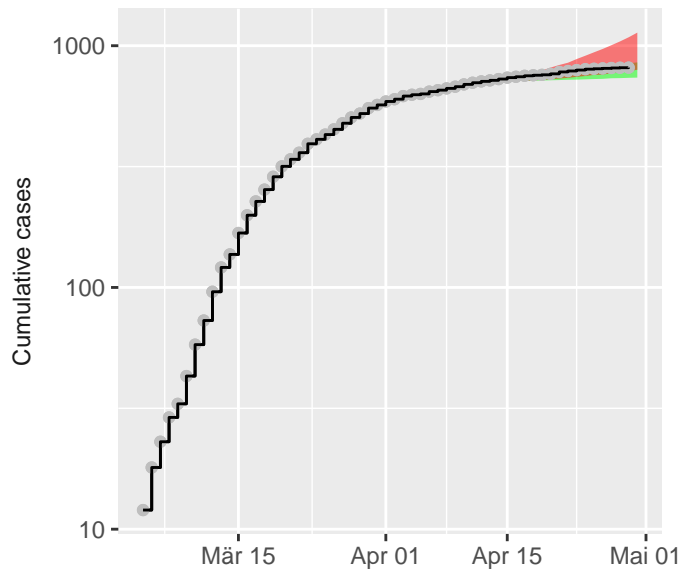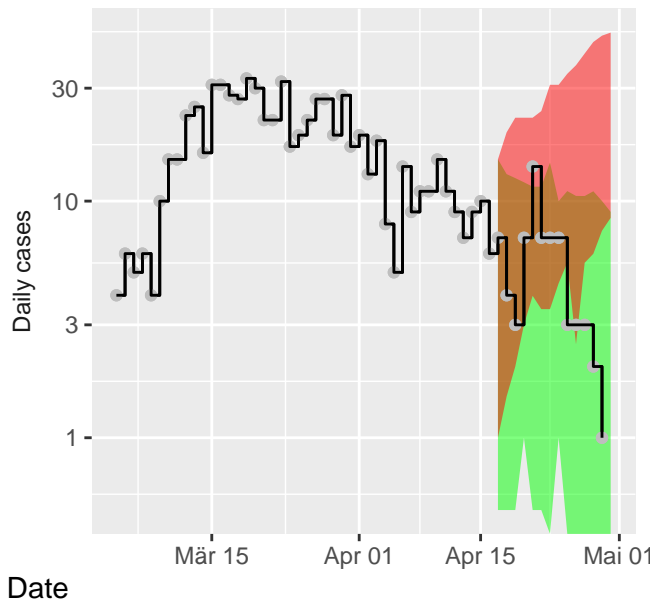

## Köln

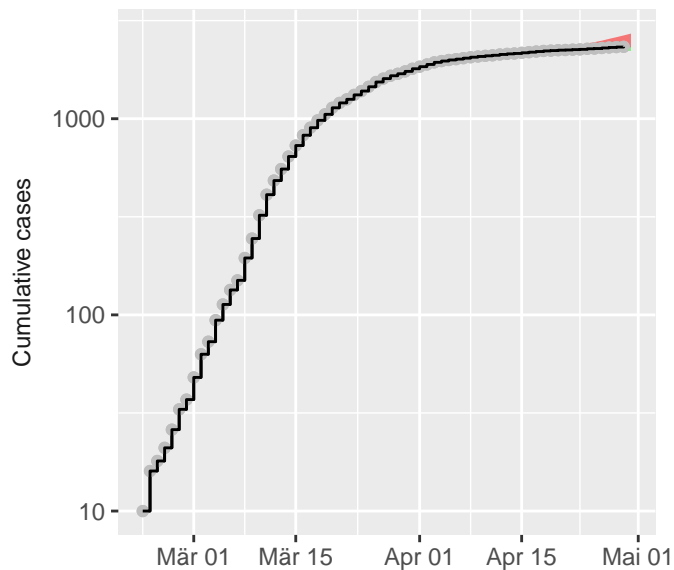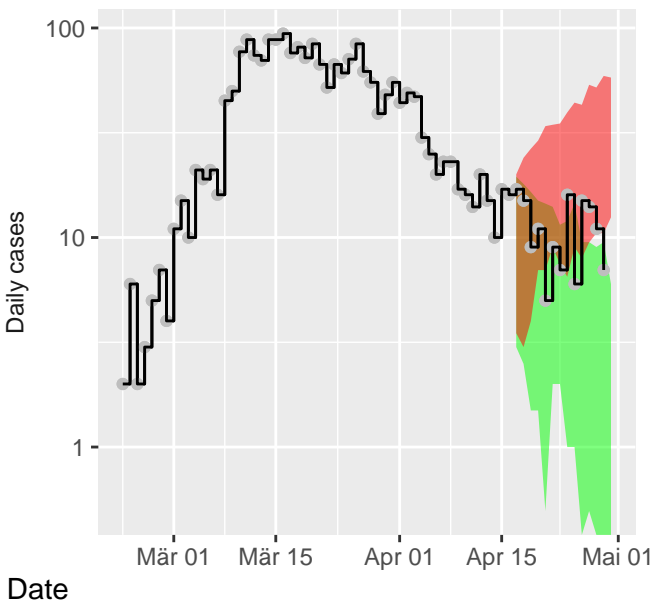

Supplement: Supplementary file 3 — Supplementary material 3 (pdf 11 KB) [file 11538_2020_834_MOESM3_ESM.pdf]

# Potsdam

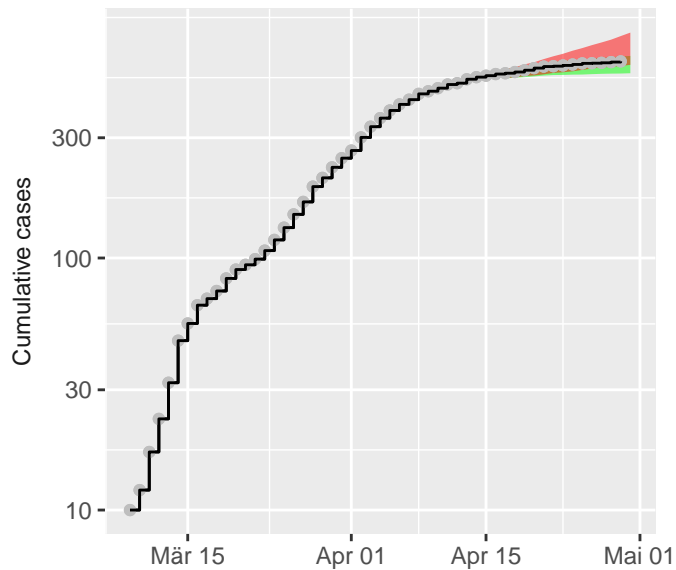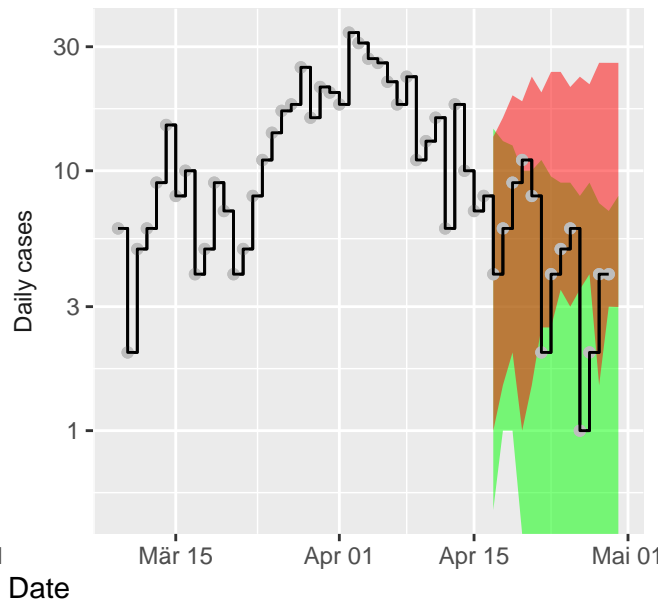

# Starnberg

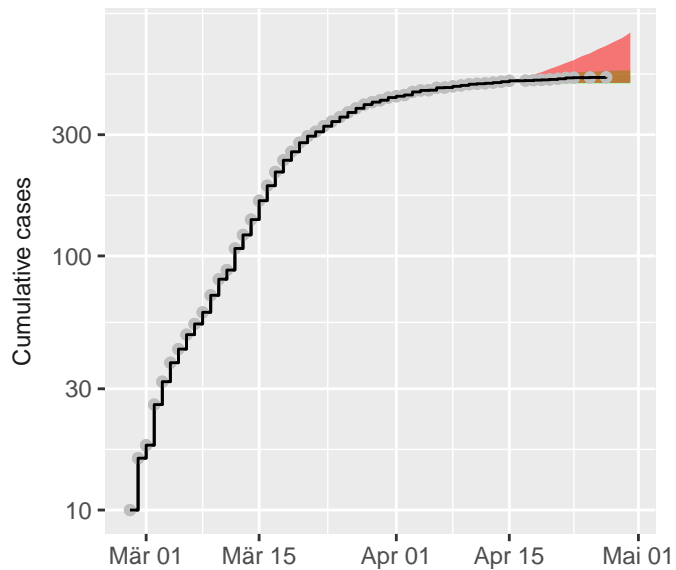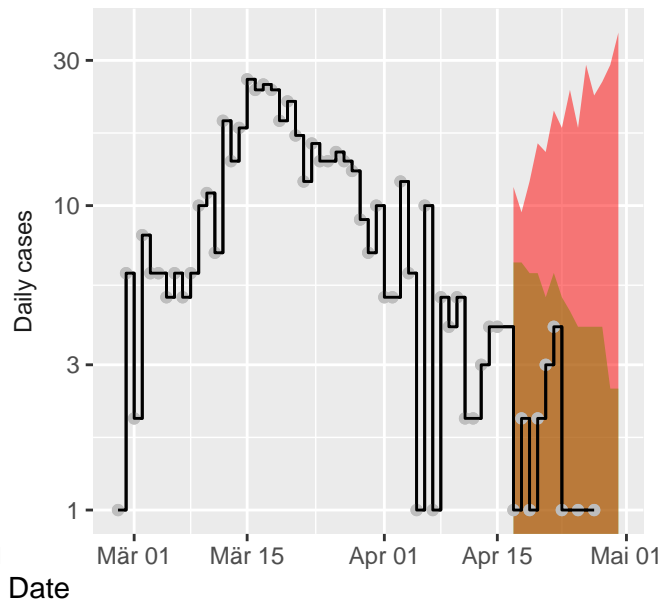

Supplement: Supplementary file 4 — Supplementary material 4 (pdf 10 KB) [file 11538_2020_834_MOESM4_ESM.pdf]

## Dresden

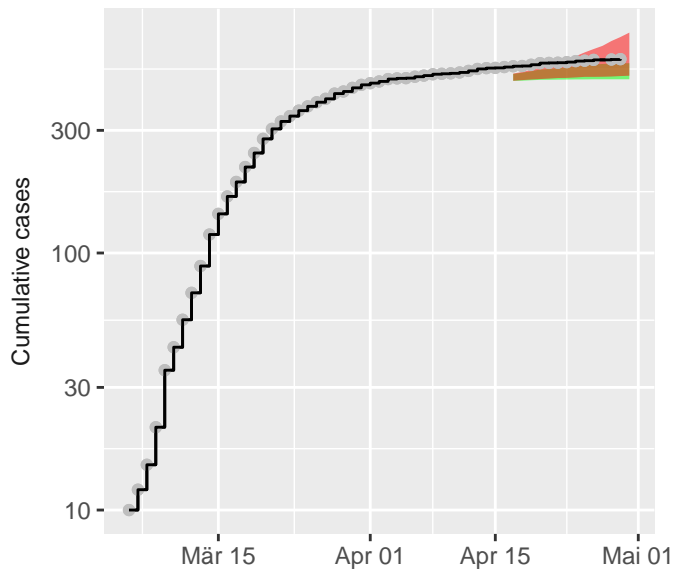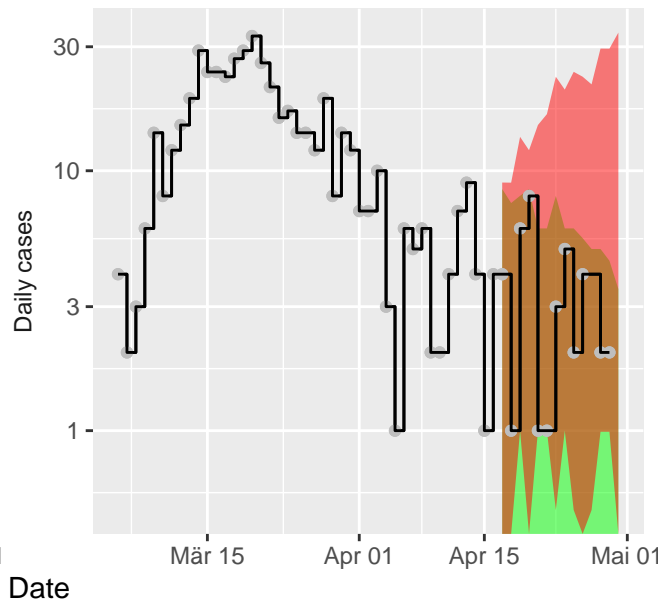

## Potsdam

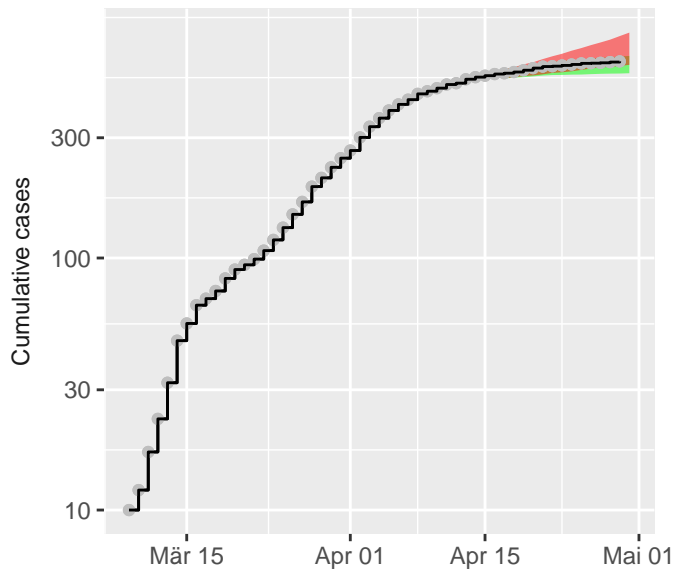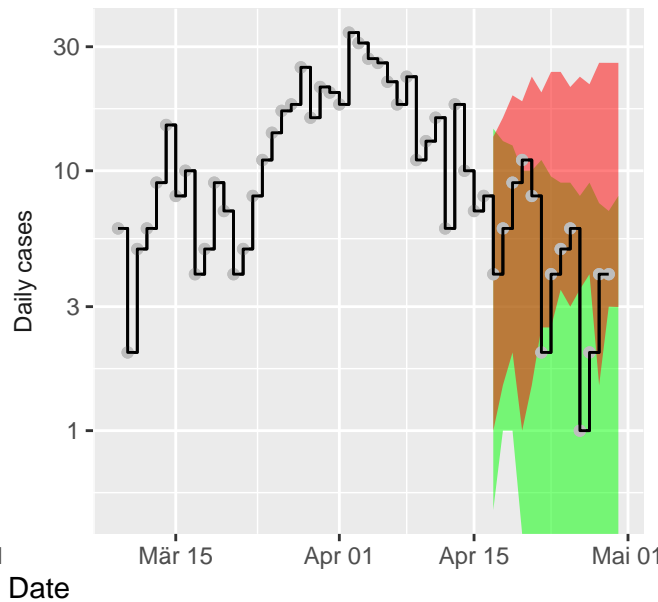

Supplement: Supplementary file 5 — Supplementary material 5 (pdf 10 KB) [file 11538_2020_834_MOESM5_ESM.pdf]

## Hohenlohekreis

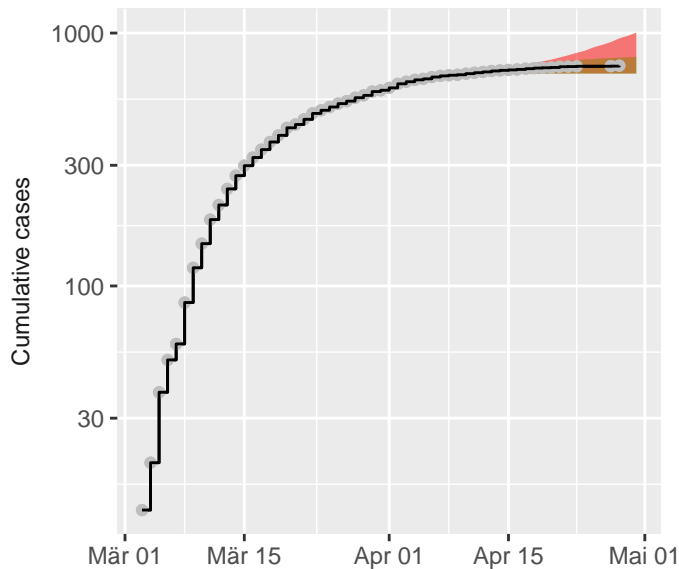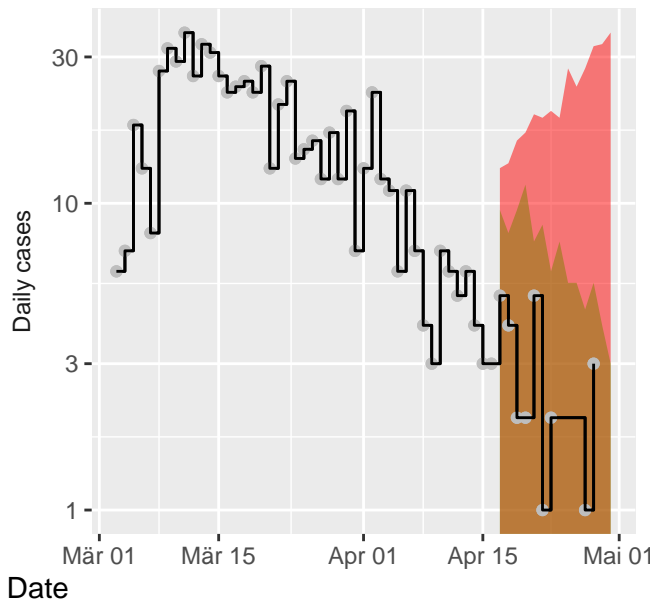

## Tübingen

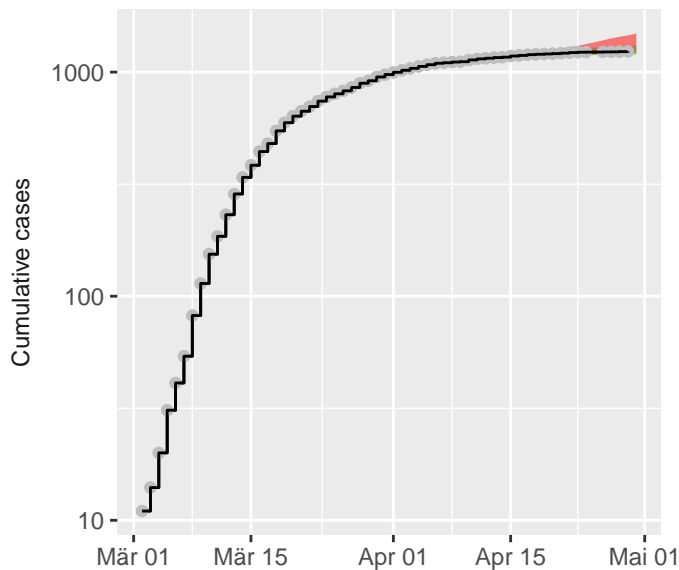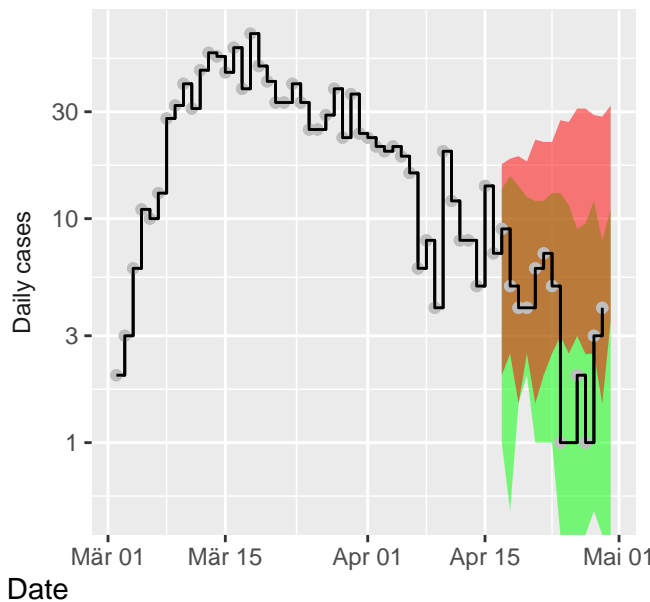

Supplement: Supplementary file 6 — Supplementary material 6 (pdf 10 KB) [file 11538_2020_834_MOESM6_ESM.pdf]

# Miesbach

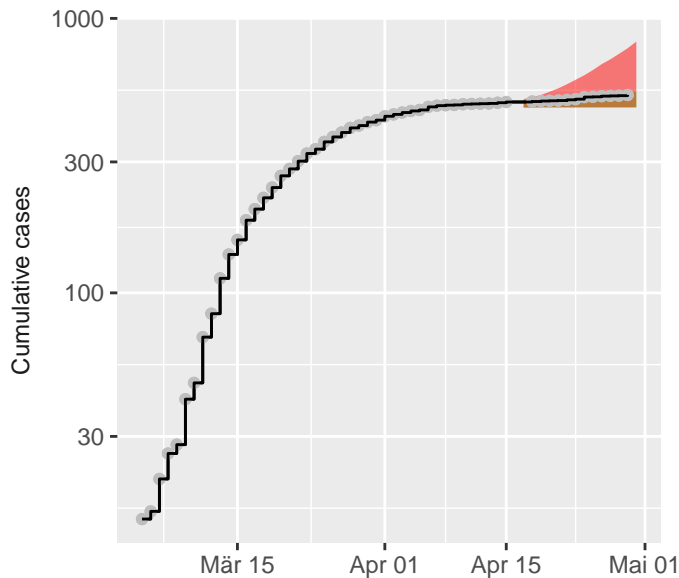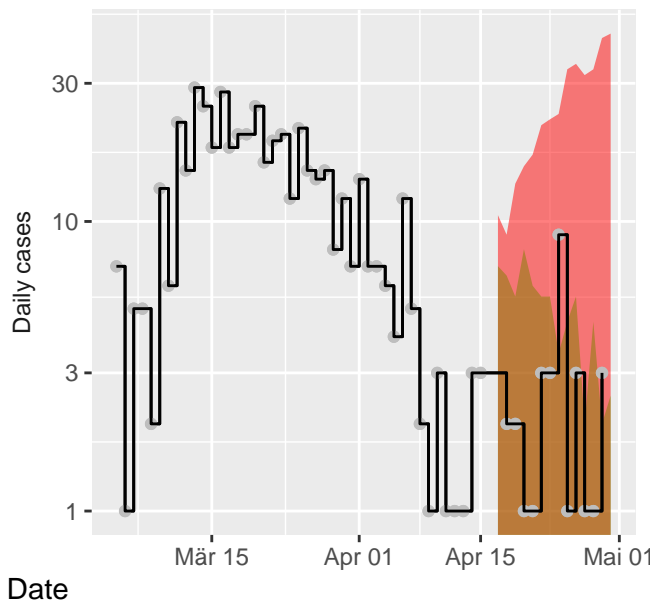

# Sigmaringen

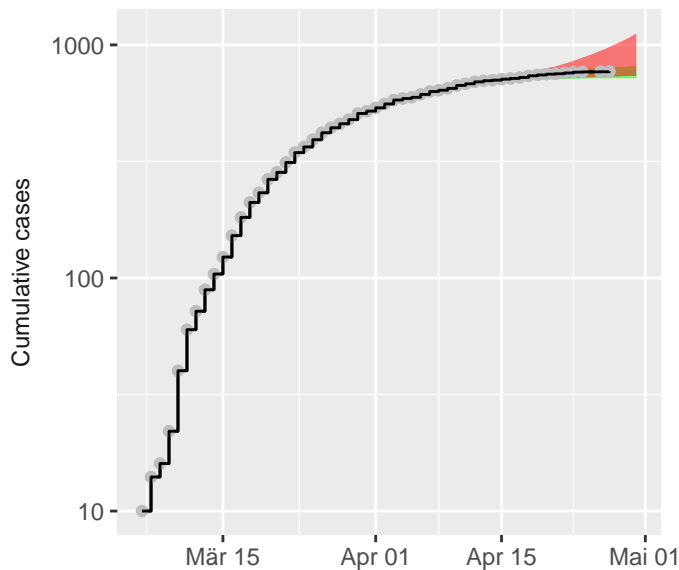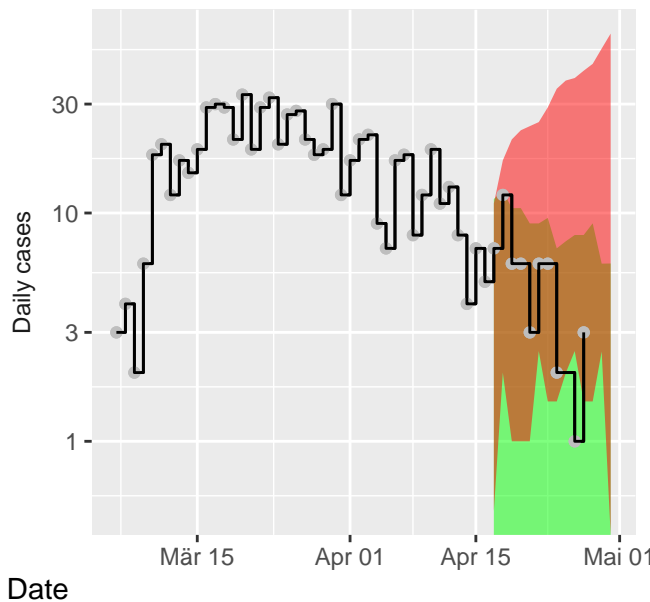

Supplement: Supplementary file 7 — Supplementary material 7 (pdf 10 KB) [file 11538_2020_834_MOESM7_ESM.pdf]
