# Supplementary material for: Sequential Data Assimilation of the Stochastic SEIR Epidemic Model for Regional COVID-19 Dynamics
Source: Bull Math Biol. 2020 Dec 8;83(1):1. doi: 10.1007/s11538-020-00834-8 (PMC7721793; doi:10.1007/s11538-020-00834-8)
Supplement: Supplementary file 8 — Supplementary material 8 (docx 10 KB) [file 11538_2020_834_MOESM8_ESM.docx]

Supplementary Information Appendix

This PDF file includes additional examples for regional modeling.

Fig. S1: Model predictions for COVID-19 after data assimilation. For details of modeling scenarios I and II see main text and Figure 6.

Fig. S2: Model predictions for COVID-19 after data assimilation. For details of modeling scenarios I and II see main text and Figure 6.

Fig. S3: Model predictions for COVID-19 after data assimilation. For details of modeling scenarios I and II see main text and Figure 6.

Fig. S4: Model predictions for COVID-19 after data assimilation. For details of modeling scenarios I and II see main text and Figure 6.

Fig. S5: Model predictions for COVID-19 after data assimilation. For details of modeling scenarios I and II see main text and Figure 6.

Fig. S6: Model predictions for COVID-19 after data assimilation. For details of modeling scenarios I and II see main text and Figure 6.

Fig. S7: Model predictions for COVID-19 after data assimilation. For details of modeling scenarios I and II see main text and Figure 6.
